# Green kiwifruit extracts protect motor neurons from death in a spinal muscular atrophy model in *Caenorhabditis elegans*

**DOI:** 10.1002/fsn3.1078

**Published:** 2019-06-17

**Authors:** Nadia Mazzarella, Ivana Giangrieco, Serena Visone, Pamela Santonicola, Jannis Achenbach, Giuseppina Zampi, Maurizio Tamburrini, Elia Di Schiavi, Maria Antonietta Ciardiello

**Affiliations:** ^1^ Institute of Biosciences and BioResources CNR Naples Italy

**Keywords:** *Actinidia deliciosa*, *Caenorhabditis elegans*, kiwifruit, neuroprotection, spinal muscular atrophy

## Abstract

Kiwifruit is considered a functional food and a good source of nutraceuticals. Among the possible beneficial effects of kiwifruit species, a neuroprotective activity exerted in rats with learning and memory impairment induced by exposure to different chemicals was reported. We sought to investigate the neuroprotective activities of kiwifruit toward spinal muscular atrophy (SMA). To this purpose, we have used a recently developed *Caenorhabditis elegans* SMA model, displaying an age‐dependent degeneration of motor neurons detected as locomotory defects, disappearance of fluorescent markers, and apoptotic death of targeted neurons. Although an anti‐nematode activity is reported for kiwifruit, it has been verified that neither green (*Actinidia deliciosa*, cultivar Hayward) nor gold (*Actinidia chinensis*, cultivar Hort 16A) kiwifruit extracts cause detectable effects on wild‐type *C. elegans* growth and life cycle. Conversely, green kiwifruit extracts have a clear effect on the *C. elegans* SMA model by partially rescuing the degeneration and death of motor neurons and the locomotion impairment. The gold species does not show the same effect. The components responsible for the neuroprotection are macromolecules with a molecular weight higher than 3 kDa, present in the green and not in the yellow kiwifruit. In conclusion, this is the first study reporting a protective activity of green kiwifruit toward motor neurons. In addition, we demonstrate that *C. elegans* is an animal model suitable to study the biological activities contained in kiwifruit. Therefore, this model can be exploited for future investigations aimed at identifying kiwifruit molecules with potential applications in the field of human health.

## 1 INTRODUCTION

Consuming fruit and vegetables is generally associated with a reduced risk of pathologies such as heart diseases, diabetes, cancer, neurodegenerative diseases, and other dysfunctions. Among fruits, green kiwifruit is widely reported as a source of nutraceuticals with beneficial effects on human health. It has been used since ancient times in traditional Chinese medicine to treat even severe diseases, such as cancer (Motohashi et al., 2002). Anticonstipation (Chang, Lin, Lu, Liu, & Liu, 2010), counteraction of cardiovascular disease (Recio‐Rodriguez et al., 2015), antibacterial (Popovic, Andjelkovic, Grozdanovic, Aleksic, & Gavrovic‐Jankulovic, 2013), and anti‐nematode (Stepek, Buttle, Duce, Lowe, & Behnke, 2005) activities have also been described.

The beneficial effects of plant foods are usually ascribed to vitamins, polyphenols, and other components with antioxidant activity contained therein (Wojdyło, Nowicka, Oszmiański, & Golis, 2017). However, some studies also report a contribution of specific protein molecules to the possible biological effects of kiwifruit. For instance, it has been demonstrated that kiwifruit has a superoxide dismutase (SOD)‐like activity (Spada, de Souza, Bortolini, Henriques, & Salvador, 2008) such that the consumption of one fruit provides more than the daily recommended dose to counteract oxidative stress (Giangrieco et al., 2016). The presence in kiwifruit of anti‐inflammatory activity has been highlighted by testing the peptide kissper (Offermann et al., 2015) in ex vivo experiments on intestinal tissues of patients suffering from Crohn's disease (Ciacci et al., 2014). A neuroprotective effect of kiwifruit species, such as *Actinidia arguta*, has also been shown in a mouse model where the fruit extract was able to improve learning and memory deficits induced by the neurotoxic compound trimethyltin (Ha et al., 2015). In addition, it has been reported that the kiwifruit *Actinidia chinensis*, cultivar Qinmei, efficiently improved learning and memory impairment induced in rats by lead exposure (Xue et al., 2017).

A possible neuroprotective activity of green kiwifruit on motor neuron (MN) degeneration caused by a genetic defect has never been investigated to date. Indeed, the testing of bioactive molecules with a potential exploitation in the treatment of specific pathologies requires the presence of appropriate disease models. The nematode *Caenorhabditis elegans* is a highly attractive model for the identification and characterization of bioactive natural molecules (Cañuelo et al., 2012; Ding et al., 2017; Parker et al., 2005; Shukla et al., 2012; Srivastava et al., 2017), also thanks to the ease and speed of methods designed to identify and characterize new disease‐related genetic mutants (Illiano et al., 2017; Di Schiavi & Andrenacci, 2013). Recently, a new *C. elegans* model useful to study the spinal muscular atrophy (SMA)‐related neurodegenerative process in vivo has been developed (Gallotta et al., 2016) and is therefore available to test the possible neuroprotective effects of natural molecules. SMA is an autosomal recessive neurodegenerative disease and is characterized by the specific loss of alpha‐MNs in the anterior horn of the human spinal cord, leading to the progressive atrophy of proximal muscles and, in severe cases, paralysis of respiratory muscles and death (Crawford & Pardo, 1996). This pathology is associated with a deficiency in the survival motor neuron 1 (*Smn1*) gene, which is ubiquitously expressed within and outside the nervous system (Lefebvre et al., 1995; Young, Le, thi Man, Burghes, & Morris, 2000). However, despite the reduction in SMN in all tissues, the hallmark of SMA is the selective loss of spinal MNs in both humans and mouse models of the disease (Burghes & Beattie, 2009; Simon et al., 2017).

The SMA model developed by Gallotta and co‐authors (Gallotta et al., 2016) displays an age‐dependent degeneration of MNs detected as locomotory defects, the disappearance of presynaptic and cytoplasmic fluorescent markers, and finally the apoptotic death of targeted neurons. Unlike other available genetic models of SMA (Di Giorgio et al., 2017; Singh, Howell, Ottesen, & Singh, 2017), this new one, taking advantage of neuron‐specific silencing of *smn‐1,* avoids the systemic effects that prevent the evaluation of the specific role of *smn‐1* in neuron survival. Therefore, this model system allows the removal of the hindrances due to the pleiotropic phenotypes and, at the same time, the exploitation of the advantages of the peculiar features of this nematode, such as the small size, rapid generation time, body transparency, and short lifespan, and allows the identification of the factors preventing the death of the MNs in vivo (de Carlos Cáceres et al., 2018).

In this study, we have sought to investigate whether kiwifruit contains protective activity toward motor neuron degeneration. For this purpose, the newly developed *C. elegans* SMA model has been exploited. In particular, the possible capacity of green kiwifruit extracts, and some components, to counteract neuronal degeneration in the transgenic nematode having the *smn‐1* silencing in a specific subclass of MNs has been analyzed.

## 2 MATERIALS AND METHODS

### 2.1 Strains

Wild‐type *C. elegans* used in this work was strain N2, variety Bristol, whereas the transgenic strains were as follows: EG1285 *lin‐15B&lin‐15A(n765) oxIs12 [punc‐47::GFP; lin‐15(+)]* X that presents green fluorescent protein (*GFP*) expression in D‐type GABAergic MNs; NA1330 *gbIs4* [p*unc‐25*::*smn‐1*(RNAi sas); *pchs‐2::GFP*] *III* that presents *smn‐1* knockdown in D‐type GABAergic MNs; and NA1355 *gbIs4 III; oxIs12 X* (Gallotta et al., 2016). Nematodes were grown under standard conditions, at 20°C ± 1.5°C on NGM (Nematode Growth Medium) agar plates seeded with dead or alive bacteria, *Escherichia coli* strain OP50 (Brenner, 1974; Gallotta et al., 2016).

### 2.2 Extract and protein component preparation from kiwifruit

Green kiwifruits, *Actinidia deliciosa* cv. Hayward, and gold kiwifruit, *Actinidia chinensi*s cv. Hort 16A, were purchased in a local market. Ninety grams of fresh fruits was homogenized using a household blender. After centrifugation at 10,400 *g*, the supernatant, corresponding to a clarified fruit juice and representing the kiwi extract, was collected, aliquoted, and stored at −20°C until use.

Individual protein components were purified from green kiwifruit following the already reported procedures for actinidin (Tuppo et al., 2008), kiwellin (Tamburrini et al., 2005), thaumatin (Palazzo et al., 2013), and kissper (Ciardiello et al., 2008). Protein concentrations were determined according to the Bio‐Rad Protein Assay Procedure, using calibration curves made with BSA.

### 2.3 Fractionation of the extract in low and high molecular weight components

Ten millilitres of green kiwifruit extract (G‐extract‐2, see below for details), prepared as described above, was subjected to ultrafiltration using Ultracel 3K Amicon Ultra filters (Millipore). The procedure was stopped when the volume of the ultrafiltrated extract, containing molecules with a low molecular weight (LMW‐extract), that is equal or smaller than 3 kDa, reached 5 ml. On the basis of observations from preliminary experiments, it was decided not to ultrafiltrate the extract further since, otherwise, some of the components would have started to precipitate and the resulting pellet would not have been easily solubilized. An equal volume, 5 ml, of unfiltered sample was collected. It contained molecules with a high molecular weight (HMW‐extract), that is equal or greater than 3 kDa. Obviously, it was conceivable that the LMW‐extract contained molecules below 3 kDa, whereas the HMW‐extract contained the components above 3 kDa. In fact, SDS‐PAGE analysis confirmed that all the protein components were in the HMW‐extract, whereas no protein bands were observed in the LMW‐extract. LMW‐extract and HMW‐extract were sterilized by filtration with 0.22‐µm membrane filters (Millipore) and diluted 1:5 and 1:10 with the liquid medium before use.

### 2.4 Influence of kiwifruit extracts on *C. elegans* life cycle and neurodegeneration

Kiwi extracts were first filtered using 1.2‐µm membrane filters (Millipore), next sterilized by filtration with 0.22‐µm membrane filters (Millipore), and then added to plates containing solidified agar with NGM to reach the final dilution. Heat‐killed or live bacteria were then added. Valproic acid (VPA) or water was added on separate plates as controls. The life‐cycle traits of wild‐type worms, after exposure to the kiwi extracts, were analyzed as previously described (MacNeil, Watson, Arda, Zhu, & Walhout, 2013). Briefly, 50 eggs were transferred onto NGM plates where the kiwi extracts had been adsorbed and a layer of *E. coli* OP50 had been seeded. Kiwifruit extract was omitted in the control plates. After 24h, the hatched eggs were counted and possible morphological defects were analyzed. After 96 hr, the counting and morphological analysis were repeated on adult animals.

The evaluation of *C. elegans* neurodegeneration and backward movement were performed as already reported (Gallotta et al., 2016). Kiwifruit extracts were added on 6‐cm plates containing NGM agar to a final dilution of 1:10 or 1:100 and allowed to adsorb for 1 day before seeding OP50 bacteria. For locomotion assay, extracts were added to 22‐mm multiwell containing NGM agar to a final dilution of 1:10, 1:20, 1:40, and 1:100, and allowed to adsorb for 1 day before seeding*. C. elegans* at L4 stage were then transferred onto the plates and removed after 24 hr. The progeny was evaluated for neuron death, neuron degeneration, and locomotion at the stage of young adult. Each experimental condition was run blindly in duplicate or triplicate. Individual protein components purified from green kiwifruit were added to liquid cultures where *C. elegans* eggs were collected and allowed to hatch and grow until adulthood, in a final volume of 50 μl. The liquid medium was prepared with dead OP50 bacteria, 9 ml of M9 buffer, and 9 μl of cholesterol.

### 2.5 Microscopy analysis

Animals were immobilized in 0.01% tetramisole hydrochloride (Sigma‐Aldrich) on 4% agar pads and visualized using a Zeiss Axioskop equipped with epifluorescence and DIC/Nomarski optics, and images were collected with an AxioCam digital camera. To discriminate dying MN fluorescence from endogenous autofluorescence, a Zeiss filter set 09 was used. This setting allowed the observation of intestinal cell autofluorescence in yellow and apoptotic fluorescence‐positive dying cells in green.

### 2.6 Statistical analysis

*GraphPad Prism* software was used for statistical analysis. The statistical significance was determined using one‐way ANOVA followed by Bonferroni post‐test or Kruskal–Wallis test, *z* statistic, or unpaired *t* tests, comparing each sample against the control. Standard error of mean was used to estimate variation within a single population in different experiments. Standard deviation was used to estimate variation within a population in a single experiment.

## 3 RESULTS

### 3.1 Kiwi extracts

**Figure 1:**
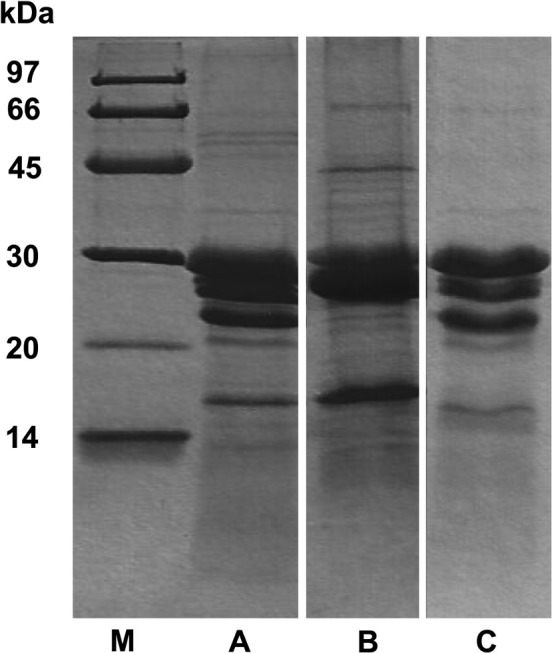
Protein pattern of green and gold kiwifruit extracts analyzed by reducing SDS‐PAGE. Twenty µg of G‐extract‐1 (lane A), Y‐ extract (lane B), and G‐extract‐2 (lane C) was loaded

Kiwifruit extracts were prepared as one batch of gold kiwifruit extract (Y‐extract) and two different batches of green kiwifruit extracts (G‐extract‐1 and G‐extract‐2). The Y‐extract had a protein concentration of 2.5 mg/ml. The protein profile is shown in Figure 1, lane B, where the major components, kiwellin (28 kDa) and kirola (17 kDa), can be observed. The first green kiwifruit extract (G‐extract‐1) had a protein concentration of 2.1 mg/ml and the protein pattern shown in Figure 1, lane A, whereas the second one (G‐extract‐2), obtained using a different commercial batch of fruits, had a protein concentration of 2.3 mg/ml and the protein profile reported in Figure 1, lane C. SDS‐PAGE highlights that G‐extract‐1 and G‐extract‐2 have a similar protein profile including the well‐known major protein components, actinidin (30 kDa), kiwellin (28 kDa), thaumatin‐like protein (24 kDa), and kirola (17 kDa).

### 3.2 Kiwifruit extracts counteract motor neuron death in *C. elegans*

*C. elegans* wild‐type larvae grown on green and gold kiwifruit extracts reached the stage of young adult in the expected time and did not present gross morphological defects, as observed in the mock control (data not shown). This observation suggests that kiwifruit does not grossly affect *C. elegans* development, and therefore, *C. elegans* can be used as a model to investigate the biological activities of kiwifruit.

**Figure 2:**
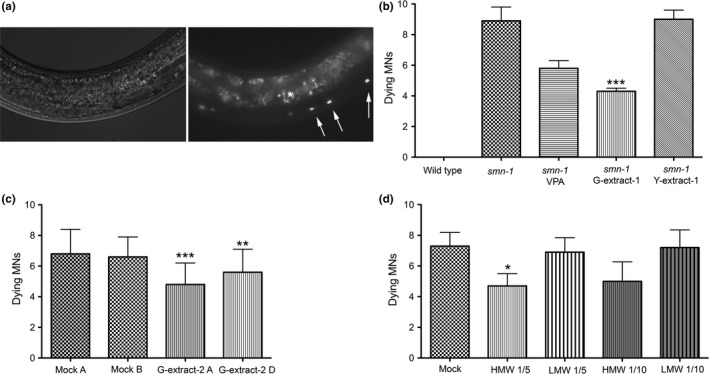
Kiwifruit effects on MN apoptotic death in transgenic *C. elegans*. Apoptotic death caused by* smn‐1* knockdown is rescued by green kiwifruit extracts. (a) Accumulation of apoptotic‐related fluorescence in dying MNs in the ventral cord in a *smn‐1(RNAi)* transgenic animal (white arrows). Images were taken with visible light (left panel) and with epifluorescence (right panel), using Zeiss filter set 09 to distinguish it from intestinal autofluorescence (asterisk). Anterior is left, and ventral is down. (b‐d) Number of dying MNs accumulating apoptotic fluorescence in control and transgenic animals under different conditions. Bars represent the average number of dying neurons per animal, from at least two independent blind experiments; *SEM* or *SD* (in c) is shown. (b) *smn‐1(RNAi)* transgenic nematodes were treated with 1mM VPA, G‐extract‐1, and Y‐extract, diluted 1:10. The extract was omitted in the control (*smn‐1*). Wild‐type animals present no MN death. The number of animals observed is 53, 277, 100, 198, and 90, respectively. ***indicates significantly different from untreated animals (*p* < 0.001 with one‐way ANOVA). (c) Transgenic nematodes were treated with G‐extract‐2, at 1:10 dilution with heat‐killed (G‐extract‐2 D) or live (G‐extract‐2 A) bacteria and compared to untreated controls (mock). The number of animals observed is 76, 63, 77, and 45, respectively. *** and **indicate significantly different from relative untreated animals (*p* < 0.001 and *p* < 0.01, respectively, with one‐way ANOVA). (d) Transgenic nematodes were treated with high (>3 kDa, HMW) and low (<3 kDa, LMW) molecular weight fractions at 1:5 and 1:10 dilutions and compared to untreated controls (mock). The number of animals observed is 86, 81, 77, 91, and 82, respectively. *indicates significantly different from untreated animals (*p* < 0.05 with Unpaired *t* test)

*smn‐1* silencing in MNs causes the death of neurons, detectable as the accumulation of apoptotic‐related fluorescence in dying MNs, whose nature has been confirmed using cell death markers and genetic mutants (Gallotta et al., 2016). This accumulation is a late event in neuronal death and independent of the origin, is a specific, late sign of the degeneration of the MNs in which *smn‐1* has been knocked down, and is never observed in negative controls. Transgenic *C. elegans* with *smn‐1* silencing in MNs grown on G‐extract‐1 displayed a significant decrease in neuronal death (Figure 2), as demonstrated by a reduction in the average number of dying MNs from 8.9, in the mock‐treated animals, to 4.3 in the treated nematodes (Figure 2b, *p* < 0.001). This rescue was higher than that obtained with valproic acid (5.8 dying MNs), a well‐established drug successfully used in cell cultures, mouse, and *C. elegans* SMA models (Brichta et al., 2003; Tsai, Tsai, Ting, & Li, 2008). In contrast, Y‐extract did not have any effect on MN death. To demonstrate the reproducibility of our findings, the experiment was repeated using an independent extract, G‐extract‐2, and again a protective effect was observed, with an average number of dying MNs of 4.8 in the treated worms and 6.8 in the controls (Figure 2c, *p* < 0.001). To exclude any involvement of bacteria metabolism in the rescuing effects, we repeated the experiment with heat‐killed bacteria and a protective effect was obtained again (5.6 dying MNs vs. 6.6 in the controls, *p* < 0.01). This result suggests that in green kiwifruit extracts, and not in the gold one, there are bioactive components with good neuroprotective activity that may cause a partial rescue of dying MNs up to 57%. The neuroprotection is not modified by bacteria metabolism, and it is independent of fruit batches.

### 3.3 Characterization of the subcomponents contained in green kiwifruit extract that counteract motor neuron death caused by *smn‐1* silencing

In the attempt to identify which components of the extracts cause the rescue of the degeneration, we fractionated the total extracts. The transgenic nematodes treated with the fraction rich in macromolecules (>3 kDa, HMW‐extract), diluted 1:5, showed an average number of dying MNs corresponding to 4.7 (Figure 2d), compared to 7.3 in the untreated controls (*p* < 0.05). Therefore, a rescue of 35% of dying neurons was recorded. In contrast, the nematodes treated with the LMW‐extract (<3 kDa), diluted 1:5, displayed a number of dying neurons (6.9) that were not statistically different from the control (Figure 2d). The same experiment was repeated using a higher dilution, 1:10, and it was observed that the HMW‐extract again counteracted the death of MNs (5.0 dying neurons) and caused a similar, albeit slightly lower, rescue (31%).

In the attempt to identify the single molecules causing the rescue of degeneration, we tested some of the protein components contained in high amounts in green kiwifruit (Figure 1), purified from the natural source, namely actinidin, kiwellin, thaumatin, and the nutraceutical peptide kissper. However, the results obtained did not show significant effects of these molecules on the MN degeneration in transgenic *C. elegans* at the concentration and growth conditions we were able to use (data not shown). These results suggest that the still elusive bioactive component/s have a molecular mass higher than 3 kDa.

### 3.4 Kiwifruit extracts promote motor neuron survival

**Figure 3:**
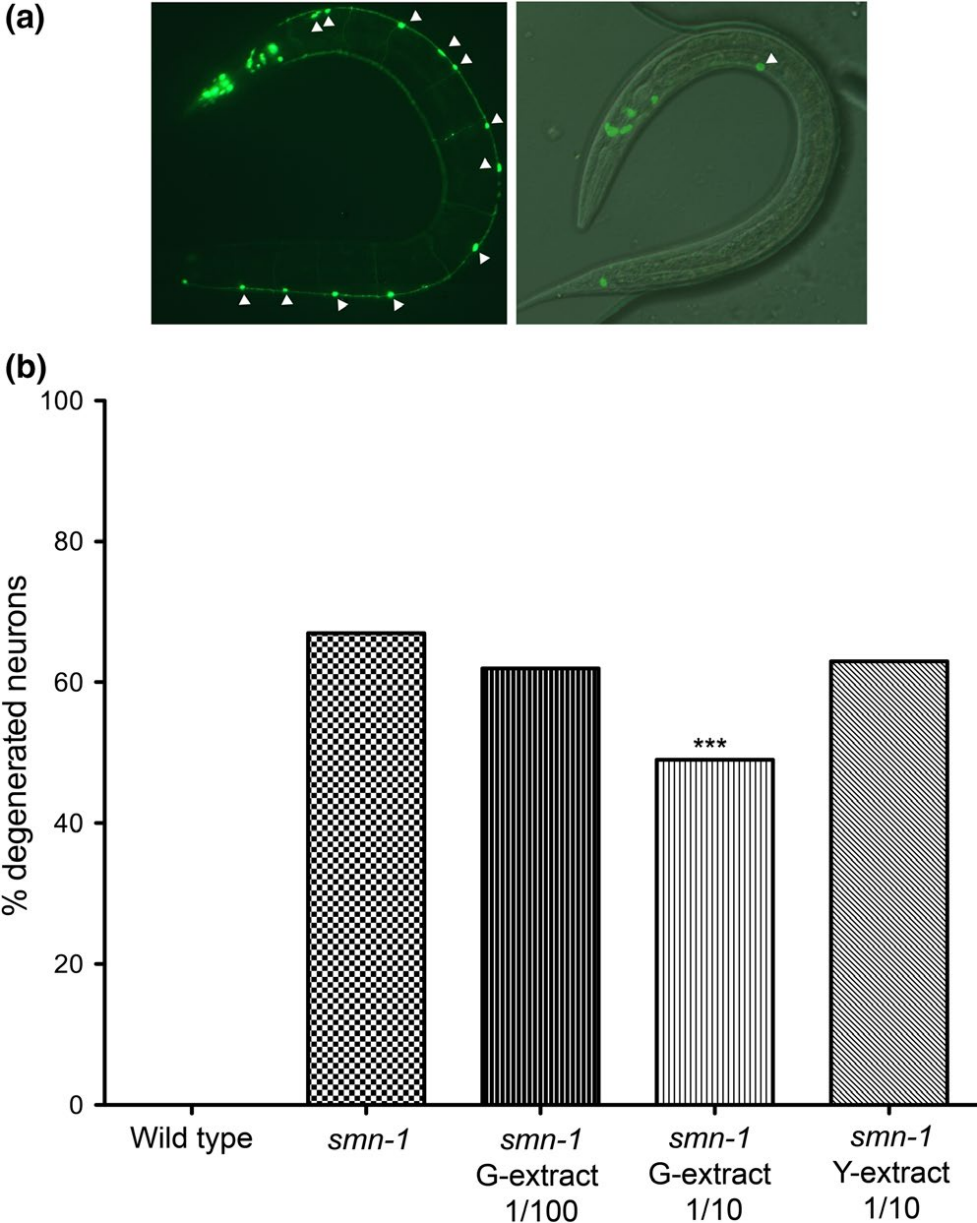
Kiwifruit effects on MN early degeneration in transgenic *C. elegans*. (a) Knockdown of *smn‐1* in MNs results in degeneration of MNs expressing GFP thanks to *oxIs12* [*punc‐47::GFP*] transgene. The animals were observed with epifluorescence (left panel) and with visible light combined with epifluorescence (right panel). The head is in the higher left part of the pictures. In control animals (left), most of the nineteen MNs are visible in the ventral cord (twelve are indicated with arrowheads). *smn‐1* knockdown (right) induces a degeneration in MNs, with only one neuron still visible (arrowhead). (b) Percentage of degenerated MNs in wild‐type, *smn‐1(RNAi)* knocked‐down animals and after treatment with G‐extract‐1 and Y‐extract diluted 1:10 or G‐extract‐1 diluted 1:100. Wild‐type animals present no MN degeneration. The extract was omitted in the control (*smn‐1*). The number of animals observed is 50, 180, 90, 90, and 90, respectively. All animals carry *oxIs12* [*punc‐47::GFP*] transgene. ***indicates significantly different from untreated animals (*p* < 0.001 with z nonparametric statistics)

After having clarified that green kiwifruit extracts cause a significant reduction in the number of dying MNs, we wondered whether these extracts were also able to promote healthy cell survival, at the dilution found to be effective to partially prevent neuronal death. In fact, the early signs of degeneration in our model are detectable in double transgenic animals with *smn‐1* silencing and GFP expression in MNs as the disappearance of GFP‐expressing neurons (Gallotta et al., 2016). We have demonstrated that healthy neurons silenced in *smn‐1* initially degenerate and do not express GFP anymore, and then accumulate apoptotic fluorescence, a late event in neuronal death, occurring after the activation of the cell death pathway genes and associated with indicators of cell death. For this purpose, we investigated the morphology of GFP‐expressing MNs, taking advantage of this double transgenic strain, after exposure to natural extracts (Figure 3). By microscopy analysis, we found a considerable improvement in neuron survival in the presence of green kiwifruit (G‐extract‐1), compared to the worms grown on the mock, as demonstrated by the reduction of degenerating MNs from 67% to 49% (Figure 3b, *p* < 0.001). No protection was obtained with Y‐extract or with G‐extract‐1 at higher dilution (63% and 62%, respectively). These results confirm, on a second and earlier phenotype caused by *smn‐1* silencing in MNs, that the green kiwifruit extract has beneficial properties in preventing the early events of SMA‐related neurodegeneration by promoting the maintenance of the wild‐type morphology of neurons, which will then results in the improved survival of the MNs.

### 3.5 Kiwifruit extracts prevent defects in locomotion

**Figure 4:**
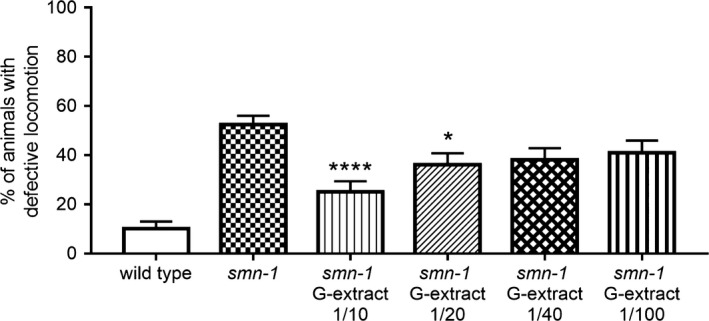
Kiwifruit effects on backward locomotion in transgenic *C. elegans*. Percentage of *smn‐1(RNAi)* knocked‐down animals with defects in backward locomotion before and after treatment with G‐extract diluted 1:10, 1:20, 1:40, and 1:100. The extract was omitted in the control *(smn‐1)* and in the wild type. The number of animals observed is 100, 200, 100, 100, 100, and 100, respectively. **** and *indicate significantly different from untreated *smn‐1* animals (*p* < 0.0001 and *p* < 0.05, respectively, one‐way ANOVA Kruskal–Wallis test). Bars represent the average percentage of animals with defects in locomotion, from at least two independent blind treatments; *SEM* is shown.

In *C. elegans*, D‐type MNs have a role in the regulation of backward movement (McIntire, Jorgensen, Kaplan, & Horvitz, 1993). The absence of *smn‐1* causes defect in backward locomotion as a consequence of the neurodegeneration (Gallotta et al., 2016). To further investigate the neuroprotective effect of kiwifruit extract, we tested the locomotion of *smn‐1* animals at the concentration that rescued the neuronal death and the neurodegeneration (1:10). We observed a reduction in the locomotion defect after treatment from 52.5% to 25.0% defective responses (Figure 4; *p* < 0.0001, one‐way ANOVA Kruskal–Wallis test). Moreover, we tested the movement at different doses (1:20, 1:40, and 1:100) to determine a possible dose‐dependent neuroprotective effect. We observed a lower but significant reduction of locomotion defects at 1:20 dose, with 36% defective responses (Figure 4; *p* < 0.05, one‐way ANOVA Kruskal–Wallis test), and no effect was observed when 1:40 and 1:100 dilutions were used, with 38% and 41% defective responses, respectively (*p* = 0.0561 and *p* = 0.2006; one‐way ANOVA Kruskal–Wallis test). These results demonstrate that the neuroprotective effect of kiwifruit extract can rescue the functional impairment observed in *smn‐1* animals, with a dose‐dependent effect.

## 4 DISCUSSION

Many biological activities have been ascribed to kiwifruit, including the anti‐nematode effects observed on the intestinal parasite *Heligmosomoides polygyrus* (Stepek et al., 2005). The experimental results, here reported, show that neither green nor gold kiwifruit cause detectable effects on *C. elegans* growth and life cycle at the tested dilutions. Therefore, the advantages of this model system can be fully exploited to study the bioactive molecules contained in this fruit.

The use of a transgenic *C. elegans* strain mimicking SMA has allowed, for the first time, the detection of a neuroprotective effect of green kiwifruit on a specific class of MNs. In fact, extracts were able to significantly decrease the apoptotic death of MNs caused by the neuron‐specific knockdown of *smn‐1*. In contrast, the gold species did not display the same property. The consistency of our findings is confirmed by similar results obtained with two independent batches of green kiwifruits. Furthermore, the effect was specifically visible only with the green species extracts for which a dose‐dependent response was demonstrated. Moreover, three consequent SMA‐related phenotypes were rescued, thus suggesting that the neuroprotection may act early in the degenerating process. The results obtained allowed the ascription of the neuroprotection activity to macromolecules with a molecular weight higher than 3 kDa, so not associated with components such as vitamins and polyphenols. This result prompted the testing of available high‐molecular‐weight‐specific components from green kiwifruit, namely the protease actinidin, kiwellin, thaumatin‐like protein, and the nutraceutical peptide kissper that was reported to be endowed with antioxidant and anti‐inflammatory activity (Ciacci et al., 2014). However, none of these molecules showed a significant neuroprotective effect, at least in the experimental conditions used. Nevertheless, we cannot exclude that, in the presence of optimal conditions, these proteins could play a role in replicating the biological activity associated with the extracts, possibly with synergistic effects.

A kiwifruit macromolecule representing a good candidate for future testing is the SOD enzyme. The literature reports different types of SOD proteins displaying neuroprotection, for instance in brain ischemia models, (Huang, Guo, Cao, Shi, & Xia, 2012; Jung, Kim, Narasimhan, Song, & Chan, 2009; Kondo et al., 1997; Polazzi et al., 2013) as well as in *C. elegans* models for neurodegenerative diseases (Liu, Banskota, Critchley, Hafting, & Prithiviraj, 2015; Luo, Zhang, Liu, & Zhao, 2011). However, although a SOD activity has been described in kiwifruit (Giangrieco et al., 2016), the purified protein is not currently available for testing.

It is also worth noting that the degree of neuronal protection was slightly different when two separate green kiwifruit extracts were used. This observation suggests that the amount of the components responsible for the biological activity may not be constant in this fruit. This is not surprising because it is well known that many factors affect the concentration of individual kiwifruit components (Ciardiello et al., 2009; Giangrieco et al., 2016).

In summary, kiwifruit does not show detectable effects on growth and life cycle of wild‐type *C. elegans*; therefore, this represents an animal model suitable to study the biological activities contained in this fruit. This study demonstrated that the kiwifruit extract has a neuroprotective role on MN survival. In fact, we observe that, unlike the gold species, green kiwifruit contains bioactive molecules with a neuroprotective function and a molecular weight higher than 3 kDa. More importantly, the functional rescue of the locomotion defects strongly supports the relevance of the approach used in this study and the impact of the results obtained. Future studies aimed at identifying these components will be focused on the screening of samples obtained by further fractionations of the fruit extract to identify the molecule(s) responsible for the biological activity. SMA represents the second most frequent genetic cause of infant mortality after cystic fibrosis (Lunn & Wang, 2008). In the last few years, many therapeutic approaches have been developed and are currently applied to SMA patients. In this context, the results here reported appear promising. Hopefully, further studies will allow the identification in kiwifruit of specific bioactive molecules that can provide a contribution to the treatment of this and other severe neurodegenerative pathologies.

## CONFLICT OF INTEREST

The authors declare that they have no conflict of interest.

## ETHICAL STATEMENT

The authors state that human and vertebrate animal testing was unnecessary in this study.
